# Mutual capacity building model for adaptation (MCB-MA): a seven-step procedure for bidirectional learning and support during intervention adaptation

**DOI:** 10.1186/s41256-024-00369-8

**Published:** 2024-07-02

**Authors:** Helen E. Jack, Ali Giusto, Alexandra L. Rose, Rukudzo Mwamuka, Imani Brown, Tarisai Bere, Ruth Verhey, Milton Wainberg, Bronwyn Myers, Brandon Kohrt, Gina Wingood, Ralph DiClemente, Jessica F. Magidson

**Affiliations:** 1 Division of General Internal Medicine, Department of Medicine, University of Washington, Harborview Medical Center, Box 359780, Seattle, Washington 325 9th Ave98104 USA; 2 Department of Psychiatry, Columbia University Irving Medical Center, New York State Psychiatric Institute, 1051 Riverside Dr, New York, NY 10032 USA; 3 Department of Psychology, University of Maryland, 4094 Campus Dr, College Park, MD 20742 USA; 4 Graduate School for Health Sciences, University of Bern, Uni Mittelstrasse, Mittelstrasse 43, Bern, 3012 Switzerland; 5 Mental Health Department, Faculty of Medicine, University of Zimbabwe, Mazowe Street, Avondale, Harare, Zimbabwe; 6 Friendship Bench Zimbabwe, 4 Weale Rd, Harare, Zimbabwe; 7 Mental Health, Alcohol, Substance Use, and Tobacco Research Unit, South African Medical Research Council, Parow, South Africa; 8 Department of Psychiatry and Mental Health, University of Cape Town, Neuroscience Institute, Groote Schuur Hospital, Observatory, Anzio Road, 1st Floor, Cape Town, South Africa; 9 Curtin enAble Institute, Faculty of Health Sciences, Curtin University, GPO Box U1987, Perth, WA 6845 Australia; 10 Center for Global Mental Health Equity, The George Washington University School of Medicine and Health Sciences, 2120 L Street NW, 6th Floor, Washington, DC, 20037 USA; 11 Mailman School of Public Health, Columbia University, 722 West 168th Street, New York, NY 10032 USA; 12 School of Global Public Health, New York University, 708 Broadway, New York, NY 10003 USA; 13 Center for Substance Use, Addiction & Health Research (CESAR), University of Maryland College Park, 1114 Chincoteague Hall, 7401 Preinkert Drive, College Park, MD 20742 USA

**Keywords:** Intervention adaptation, Cultural consciousness, Global health, Partnership, Bidirectional learning, Capacity building

## Abstract

Global health reciprocal innovation emphasizes the movement of technologies or interventions between high- and low-income countries to address a shared public health problem, in contrast to unidirectional models of “development aid” or “reverse innovation”. Evidence-based interventions are frequently adapted from the setting in which they were developed and applied in a new setting, presenting an opportunity for learning and partnership across high- and low-income contexts. However, few clear procedures exist to guide researchers and implementers on how to incorporate equitable and learning-oriented approaches into intervention adaptation across settings. We integrated theories from pedagogy, implementation science, and public health with examples from experience adapting behavioral health interventions across diverse settings to develop a procedure for a bidirectional, equitable process of intervention adaptation across high- and low-income contexts. The Mutual capacity building model for adaptation (MCB-MA) is made up of seven steps: 1) Exploring: A dialogue about the scope of the proposed adaptation and situational appraisal in the new setting; 2) Developing a shared vision: Agreeing on common goals for the adaptation; 3) Formalizing: Developing agreements around resource and data sharing; 4) Sharing complementary expertise: Group originating the intervention supporting the adapting group to learn about the intervention and develop adaptations, while gleaning new strategies for intervention implementation from the adapting group; 5) Reciprocal training: Originating and adapting groups collaborate to train the individuals who will be implementing the adapted intervention; 6) Mutual feedback: Originating and adapting groups share data and feedback on the outcomes of the adapted intervention and lessons learned; and 7) Consideration of next steps: Discuss future collaborations. This evidence-informed procedure may provide researchers with specific actions to approach the often ambiguous and challenging task of equitable partnership building. These steps can be used alongside existing intervention adaptation models, which guide the adaptation of the intervention itself.

**Supplementary Information:**

The online version contains supplementary material available at 10.1186/s41256-024-00369-8.

## Background

The movement of ideas or technology from low- and middle-income countries (LMICs) to high-income countries (HICs) has been called “reverse innovation” [1]. This concept, however, echoes the extractive and hegemonic relationship that has traditionally characterized the relationship between HICs and LMICs [2]. “Development aid” is similarly unidirectional, with knowledge and resources flowing from HICs to LMICs, often without respect for the strengths of LMIC communities [3]. In contrast, “reciprocal innovation” [4, 5] is the idea that people from different settings have similar challenges and can share ideas and resources across settings [6]. While there is a growing literature on the theoretical importance of reciprocal innovation [2], there have been few examples of its operationalization [5, 7–10] or structured procedures to guide practice.

As the field of global health endeavors to be more equitable and anti-colonialist [11–13], we need strategies that foster collaboration and acknowledge that many health challenges are truly global [14]. We cannot ignore vast resource differences between HICs and LMICs, which have led to inequity in access to healthcare services, but also must acknowledge the inequity in healthcare within HICs. These resource differences and inequities are rooted in historical injustice (e.g., slavery, colonialism, exploitation for natural resources). Resource redistribution must be coupled with a push for LMIC leadership and relationships between HICs and LMIC collaborators characterized by mutual respect. HIC researchers who look for LMIC partners for guidance on addressing problems they face “at home” may approach work in LMICs with greater humility [13]. Yet as implementing evidence-based interventions from LMICs in HICs increases, there is a need to be “guardrails” to ensure that the LMIC originators are included, compensated, and acknowledged.

We propose a procedure for a mutual capacity building approach when adapting interventions from a low-income context to a high-income context or vice-versa. Cultural adaptation is a first step in the implementation and scale-up of evidence-based interventions, as they must be tailored to the needs and resources of the new setting prior to widespread implementation. Adaptation may be particularly important in behavioral health, as how mental health conditions present and are best addressed may be specific to a context and culture [15–17]. The procedure we propose could be used by researchers, implementers, program developers, or practitioners [18]. Although partnership building cannot be entirely driven by a set of steps to follow, providing steps for building such partnerships could prompt conversations, provide concrete activities that funders could support, and lend clarity to approaching the amorphous challenge of mutual capacity building.

## Intervention adaptation: a space for LMIC and HIC collaboration

Evidence-based health interventions, particularly behavioral interventions, are frequently adapted from the setting in which they were initially developed, often translated, and tested for use in a new setting or population [19]. Adaptation facilitates scale-up of an evidence-based practice, while being responsive to the needs and cultural practice of new communities or populations [15, 20]. In many cases, interventions initially developed in HICs are adapted for LMICs and for delivery by non-specialist or lay providers [5, 16, 21]. For instance, Life-Steps, a brief problem-solving and motivational interviewing based approach to improve adherence to antiretroviral therapy for people living with HIV, was initially developed in the United States [22], but has now been adapted for use in Zimbabwe [23] and South Africa [24]. Increasingly, however, interventions from LMICs are also being adapted for HICs. For instance, the Friendship Bench [25], a lay health worker-delivered intervention for depression care in Zimbabwe based on problem solving therapy, was adapted for implementation in New York City [26]. Intervention adaptation has traditionally been focused on a single setting, rather than rooted in mutual learning: people in Setting B adapt an intervention from Setting A to fit their context and culture, but people from Setting A may not take lessons from Setting B’s adaptation to their setting.

There are many existing models to guide intervention adaptation, such as the widely used Assessment, Decision, Adaptation, Production, Topical experts, Integration, Training, and Testing (ADAPT-ITT), developed for HIV interventions [20], mental health Cultural Adaptation and Contextualization for Implementation (mhCACI) developed for psychological and other mental health interventions [18], and PRISSMA which centers culturally-informed adaptation [27]. These and other models typically provide a set of steps or principles that researchers and practitioners can use to tailor an intervention to a new setting or population [27, 28]. What has been lacking, however, is a clear procedure for how to ensure collaboration during adaptation processes that span historical and current power imbalances.

## Development of a procedure for mutual capacity building for intervention adaptation

Our goal was to develop an evidence-informed procedure for mutual capacity building during intervention adaptation. We drew from both relevant theoretical foundations and our team’s experiences working across HIC and LMIC settings, including several pilot studies. To develop the Mutual Capacity Building Model for Adaptation (MCB-MA), we first analyzed a series of in-depth case studies describing behavioral health interventions that had been collaboratively adapted and implemented in both HICs and LMICs. Through the case studies, we highlighted strategies these projects used to facilitate collaborative work (full case studies and strategies described elsewhere: [29]). We also piloted a mutual capacity building process for sharing study results and merging data across an LMIC and HIC that were implementing a similar peer-delivered behavioral activation intervention [10]. Our team then discussed examples of where collaboration had broken down or perpetuated inequities, which highlighted key gaps that needed to be filled through a more structured mutual capacity building process. To address those gaps, our international team proposed a series of steps, then refined them through smaller group discussions, grounded in theory and lived experience of trying to address the challenges in equitable partnerships. In Supplementary Table 1, we highlight several relevant theories that guided us, including Freirean participatory dialogue, community-based participatory research, theory of change, and several models from implementation science [30–32]. In Table 1, we briefly describe illustrative examples and counterexamples that informed our work, drawn from our experiences conducting studies and implementing behavioral health interventions in the United States, Zimbabwe, South Africa, Mozambique, Brazil, and Australia, among other countries. While the results of these and other projects conducted between HICs and LMICs have been published [33–38], these publications often lack granularity on how the partnership was formed and maintained, or the results are published separately without attention to lessons learned between sites.

**Table 1:** Examples of mutual capacity building

**Example**	**Corresponding MCB-MA step**
Two students, one from the United States and one from South Africa, did a mentored research project in which they jointly analyzed a qualitative data set that included transcripts from both Cape Town, South Africa, and Baltimore, Maryland, United States. Both received a stipend for their time and will be joint authors on the publication. They worked together, sharing both knowledge of their settings and their methodologic expertise.	Sharing complementary expertise
A single United States-based research group developed a peer-delivered behavioral intervention for patients with substance use disorder that they implemented in both the United States and South Africa. They simultaneously adapted the intervention based on in-depth qualitative research with patients and key stakeholders in each setting and piloted the adapted intervention in each site. They involved the peer recovery specialist from the United States in developing the peer role in South Africa and talking with peers from South Africa about the peer recovery specialist role, which is well-established in the United States and less common in South Africa for substance use. The team in South Africa developed a step-by-step manual on how to deliver the intervention that the United States team subsequently adopted. The US-based peer recovery specialist is also now conducting a biweekly peer group that brings together US- and South Africa-based peers working across the team’s trials to share experiences. The peers from both sites are respected members of the team who play formative roles in adapting the intervention and its implementation and are engaged in bidirectional learning in an ongoing way.	Reciprocal training Sharing complementary expertise
A group of researchers and policymakers have adapted a stepped care model that was developed in Mozambique for use in New York City. When new staff were initially trained in the stepped care model, Mozambican trainers conducted some of the trainings. The project specifically sought to employ people in both New York and Mozambique who were bilingual (English-Portuguese) to facilitate cross-site communication and learning. To do this, they often hired people in the Brazilian or Mozambican diaspora living in New York, individuals who also brought important lived experience to their roles in the project. When onboarding new staff in New York, project leadership start with a history of how the stepped care model they use was developed in Mozambique. They consider this historical context to be important in creating a culture in which the Mozambican contributions are acknowledged and respected.	Sharing complementary expertise Reciprocal training
*Counterexample –* A research group in LMIC collaborated with researchers from HIC to dramatically adapt a therapeutic model to meet local needs and resource limitations. They then tested this intervention and showed it was effective. This made their adaptation attractive to a public health group in HIC, who wanted to take their adapted model and further adapt it for their setting. The LMIC group was not involved in the adaptation for the HIC, and data sharing was complicated by the HIC adapting group’s policies.	Mutual feedback Reciprocal training
A group in an upper middle-income country (South Africa) developed a community health worker delivered intervention for depression that was subsequently adapted by a group in a low-income country (Ethiopia). The South African group was involved as consultants in the adaptation process and provided training to the Ethiopian team. The Ethiopian team developed strategies for overcoming literacy and workforce barriers to implementation that were incorporated into the originating team’s implementation training manual in South Africa.	Sharing complementary expertise Mutual feedback
A group in an upper middle-income country (South Africa) developed a lay health worker delivered intervention for adolescents that was subsequently adapted for use in a high-income country (Australia). The South African developer led the adaptation for Australia. The South African supervisor supported some of the training. The Australian adaptation included a youth advisory group that contributed to lay health worker training and intervention co-adaptation. This innovation has been shared with the team in South Africa.	Reciprocal training Sharing complementary expertise Mutual feedback
*Counterexample –* A group in a lower-income country partnered with a group in an HIC that had developed a psychological intervention because the lower-income country group wanted to adapt the intervention for their setting. The lower-income country expected and hoped that the HIC group would train clinicians and supervisors, who would subsequently train the lay facilitators. They felt that local clinicians were best placed to train lay facilitators because they were fluent in the local language and understood the local culture and context. Instead, the HIC group trained the lay facilitators in a single training, which also included the local clinicians and supervisors. The local clinicians and supervisors felt that some of the lay facilitators had to be excluded from participating in the intervention because they did not speak or understand English sufficiently to participate in the training. The HIC group wanted the training and intervention to be conducted in English, in part, to facilitate evaluation of fidelity to the original intervention content across different countries.	Reciprocal training

## Mutual capacity building model for adaptation (MCB-MA)

Here, we consider an *intervention* to be a specific treatment protocol or treatment package (e.g., number of sessions, delivery modality, interventionist, content), rather than a broader therapeutic modality (e.g., cognitive behavioral therapy). We consider *adaptation* to be a process of modifying an intervention for a new setting following an *existing adaptation framework*, such as ADAPT-ITT [20] or mhCACI [18]. We assume that all adaptation frameworks involve some formative work to develop an understanding of the new context, development of an adapted intervention protocol, and pilot testing or initial implementation of the adapted intervention in the new setting.

MCB-MA is predicated on the idea that a group that either developed the intervention or is well-established in using it (*“originating group”*) and the group that is adapting the intervention for their setting (*“adapting group”*) collaborate in a bidirectional partnership. The originating group brings expertise on the intervention and the adapting group expertise on the context and culture where it will be implemented [17, 39]. Either the LMIC or HIC group can be the originating group. Importantly, engagement in MCB-MA is *premised on the recognition of a common challenge*. The magnitude of the problem may differ between settings; although lack of access to specialist and other mental health services may be a challenge in many US and Australian cities, for instance, there remains far less access in LMICs like Zimbabwe, Mozambique, and South Africa [40].

Implementing this procedure in its entirety or in the prescribed sequence of steps may not be feasible or useful in every situation. We propose this as a starting point for conversations about mutual learning and a procedure from which groups could engage with select steps.

## MCB-MA steps

**Fig. 1:**
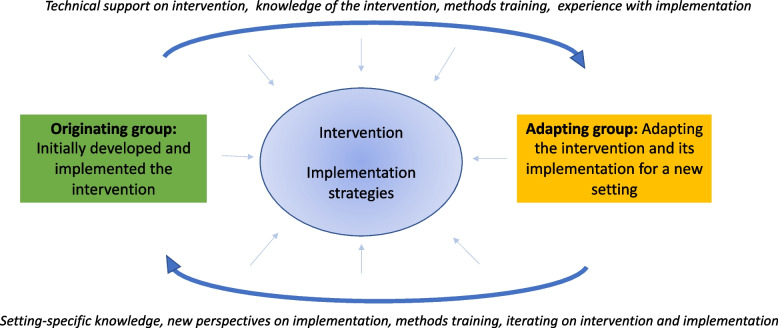
MCB-MA model

MCB-MA has three broad phases, which are summarized in Table 2; the overall model is displayed in Fig. 1.

**Table 2:** Mutual capacity building model for adaptation (MCB-MA)

		**Activities**
**Partnership building – *Prior to intervention adaptation***		
1	Exploring	- Having initial conversations about the interest each partner has in engaging in the project and the project scope, without a binding commitment
2	Developing shared vision	- Agreeing on common goals, timeline, and markers for success - Discussing power sharing and imbalances in power and privilege within the partnership - Discussing existing intellectual property and any limits to the scope of potential adaptations to the existing intervention - Selecting adaptation framework
3	Formalizing	- Developing agreements around resource and data sharing, intellectual property, developing a Memorandum of Understanding (MOU), establishing clear roles, setting standards for meetings and communication, planning and resource allocation for capacity building and reciprocal training, and planning for dissemination of findings (including authorship) - Developing a plan for building cohesion as a team
**Partnership sustaining – *During intervention adaptation and pilot testing***		
4	Sharing complementary expertise	*Regardless of adaptation framework selected, adaptation will necessitate formative work in the new setting to understand the context and development of a protocol for an adapted intervention.* - Originating group acts as consultants, providing input on intervention components and core elements as the adapting group develops the adapted protocol. - Partners identify complementary methodologic strengths and weaknesses, such as use of the adaptation framework, ways of evaluating interventions, academic writing and publishing, implementation science, or community engagement; partners identify opportunities for “just in time” teaching as the intervention is being adapted and piloted.
5	Reciprocal training	*Regardless of adaptation framework selected, implementing the adapted intervention will necessitate training practitioners, an activity that can be supported by the originating group* - Originating and adapting groups arrange who will lead the training, balancing the adapting groups’ expertise on the setting with the originating groups’ expertise in the intervention. Originating group highlights lessons learned and potentially implementation pitfalls from their prior work. - Throughout the training, originating group reflects on changes in the intervention or its implementation that may be useful in their setting.
6	Mutual feedback	- Following initial adaptation, training, and piloting of the new intervention, originating and adapting groups have a series of meaningful touch points (meetings, calls, electronic communication) to share the protocol for the adapted intervention, training outcomes data, and pilot outcomes data, respectively. These meetings can also include external stakeholders, including policymakers or community members. This may focus on implementation outcomes. - Adapting group solicits feedback from the originating group on how they might iteratively improve on these outcomes. - Originating group solicits feedback from adapting group on challenges they are having with the intervention, which may inform subsequent modifications to the intervention or its implementation.
**Partnership longevity – *Following intervention adaptation and pilot testing***		
7	Consideration of next steps	- After the adapted intervention is piloted, partners discuss whether they would like to continue to collaborate after intervention adaptation has been completed.

### Phase 1: partnership building

The goal of this phase is to agree to collaborate to adapt an intervention and develop the formal and informal structures to govern sharing of knowledge, power, and other resources during adaption.

#### Step 1: Exploring a potential partnership and the new setting

This step provides a space for open discussion about whether both groups want to engage in shared work around a common problem and time for appraisal of the new context. In initial conversations, the groups can develop shared understanding of the mutual challenge or challenges that they hope to address with the intervention; share information about their context, strengths, and needs [27]; and discuss interest in taking a mutual capacity building approach. Either group can initiate the conversation about engaging in the process of forming a partnership to adapt an intervention for one or both settings. Ideally, this conversation would occur before a project is funded so that both groups have a say in formative project development and budgeting [41]. This foundational work, however, may require funding, particularly to support LMIC partners.

Additionally, while each partner may understand their own health system and the context of the challenge they are trying to address, they can share this context with the other potential partner and conduct a situational appraisal to fill any knowledge gaps, laying the foundation for contextually appropriate future work. This appraisal may be particularly important if the adapting group is working within their own country but in an region or health system where they do not live or work, such as a rural area. All members of the adapting team, including both frontline staff and team leaders, should be familiar with existing services, local needs, cultural norms, and policies and regulations affecting the place where they will be adapting the intervention. A shared contextual understanding is important to establish up front, as it may affect what intervention the teams choose to adapt and how they go through the adaptation and implementation process.

#### Step 2: Developing a shared vision

This step allows both groups to make their adaptation plans more concrete, mixing discussion of logistics with broader conversations about distribution of power and resources.

The groups may already come to the process with an intervention in mind but should explicitly agree on the intervention to be adapted. Based on the contexts, resources available, situational appraisal, and intervention itself, the groups can select an intervention adaptation framework [42] and discuss the adaptation process:

1. Values: Do the groups agree on goals for intervention adaptation and principles that will govern the process [30, 43]?
2. Language: In what language will the intervention be delivered in the adapting setting, and what are translation needs? If the groups have different native languages, in what language will meetings take place? Can some team activities take place primarily in the non-dominant language?
3. What are “core” elements of the intervention that must be preserved and cannot be adapted across settings [44]? What are “deep structure” adaptations that may be necessary to fit within the cultural values and health or social system in the new setting [17, 45]?
4. Timeline: Over what time period does the adapting group propose to adapt, pilot, and implement the intervention [32]?
5. Planning for feedback: What data does the adapting group plan to collect during the adaptation and pilot process? Can those data be shared with the originating group [19, 46]?
6. Strengths and areas for growth: The groups can share the areas where they feel they have human or resources, knowledge, or skills and areas where they lack these. The groups can work to identify opportunities for mutual support and learning.
7. Power, privilege, and resources: The groups may have imbalances in resources, formal training, or levels of historical advantage. This can be discussed in initial conversations so that the groups can share how they will create ways of working to mitigate these. Very concretely, the groups may discuss how project resources will be directed to not further entrench advantage (e.g., pay parity between sites; ensuring that indirect costs are held by the lower resource site to contribute to institutional development) [31].
8. Building team unity: Discuss how to come together and build unity as a project team. This could involve structured team-building discussions and exercises or more informal social events or gatherings. The relationships within the team are foundational for the success of the adaptation process and may necessitate conscious effort to develop team culture [27].

#### Step 3: Formalizing

Often, projects require grants, contracts, data sharing agreements, or memoranda of understanding. This will look different for every project and set of institutions. This step ensures that those formal processes are not addressed until there have been some more fundamental conversations about partnership.

The group can discuss what they need to formalize the partnership and promote clear expectations, roles, and understanding across groups. This could include rights to use or adapt the intervention materials, data sharing agreements, ethical approvals, publication agreements, exchange of existing monetary resources between the two groups (for instance, the adapting group purchasing rights to the intervention materials or the HIC group supporting adaptation in an LMIC given deep resource inequalities and greater access to funding for HICs), or individual or joint funding applications to support the project. Pragmatically, the groups might consider applying for one of the growing number of funding opportunities for planning or partnership building [47] and structuring subcontracts to allow for flexible reallocation of funds between sites given the iterative nature of the MCB-MA process.

### Phase 2: partnership sustaining

The goal of this phase is to adapt the intervention in a way that includes both groups in complementary roles.

#### Step 4. Sharing complementary expertise

Throughout the process of adaptation, the two groups can identify complementary areas of expertise, whether that is the intervention (originating group is expert), the setting (adapting group is expert), or specific methodologies or techniques (either).

In most adaptation frameworks, the adapting group does the formative work in their setting. The adapting group will also need to familiarize themselves with the intervention itself. The originating group can act as consultants, providing input on intervention components and core elements as the adapting group develops the adapted protocol. This may help ensure fidelity to mechanisms of action and other core elements of the original evidence-based intervention [48]. The originating group may also share lessons learned from their efforts to implement the intervention, helping the adapting group consider implementation from the outset [44]. This is also, however, a time during which the originating group can learn from the adapting group’s process and changes to the intervention, providing ways that they could improve on the intervention or its implementation in their setting.

Building on their discussion of strengths and areas for growth, the groups could identify ways to support each other in their adaptation of the intervention. For instance, they may identify complementary strengths and weaknesses in the methodologies used to adapt the intervention, such as qualitative interviewing, design of pilot trials, community engagement, writing, implementation science, or community engagement [27]. This could create opportunities for “just in time” teaching as the intervention is being adapted and piloted.

#### Step 5. Reciprocal training

This step ensures that the originating group is involved in training the interventionists in the new setting in the adapted intervention. Currently, this has often happened when the intervention is being adapted from an HIC to LMIC (HIC partners do the training) but less common when an intervention is adapted from an LMIC to HIC (LMIC partners may not be invited to HIC to provide training). This step is a reminder that bidirectional training should occur in both circumstances.

Given the experience that the originating group has in delivering the intervention, they may be able to train the adapting group in the intervention, whether training only the research team or directly training the new interventionists. It is likely not sufficient to have only the originating group train the interventionists, as they will have limited experience in the language, population, and setting. Being involved in training would give the originating group an opportunity to witness the implementation of the intervention in a new setting, possibly informing their own implementation strategies. Direct involvement of the originating group in interventionist training may not be feasible or desired in all contexts but can be considered as a possible opportunity for joint learning. Involvement of people with lived experience of the target health conditions is vital both as trainers and recipients of training [49, 50].

#### Step 6: Mutual feedback

This step provides a space for the groups to share implementation or effectiveness outcomes of the adaptation process and reflect on lessons learned for both sites.

While bidirectional feedback will ideally be happening throughout the process, there should be particular focus on mutual feedback and evaluation of the process after initial implementation and effectiveness results are available. The adapting group could share the data they collected during the adaptation process and initial implementation and solicit feedback from the originating group on how to improving the intervention or its implementation [19, 46]. The originating group could then reflect on lessons that they are taking away for their own setting from the adaptation and implementation of the intervention in the new setting. The groups can also use these meetings as an opportunity to discuss how adaptations might affect clinical or implementation outcomes, share ideas about future evaluation [51], and reflect on or formally evaluate the MCB-MA process itself.

### Phase 3: partnership longevity

The goal of this phase is to make time to discuss next steps for any future joint work.

#### Step 7: Consideration of next steps: exploring continuity between the adaptation partners

At the close of the intervention adaptation, partners can discuss what comes next, including subsequent research or implementation steps. This is also an opportunity to share results of any formal or informal evaluation of the MCB-MA process that the teams did. This discussion can also include whether they would like to continue collaborating and, if so, in what form. This could take a similar format to “Exploring” and could start another cycle of MCB-MA components. It is also an opportunity for either group to discontinue the partnership and renegotiate the terms of collaboration.

## Future directions and opportunities for changing how we work

We have outlined a procedure for incorporating mutual capacity building into intervention adaptation: MCB-MA. Our goal was to provide researchers and practitioners with a set of steps that they can use to guide bidirectional adaptation processes. We hope this may help increase the frequency and equity of reciprocal innovation in global mental health. Bidirectional learning will take place within intrinsically unequal and flawed systems and hope that the mutual capacity building process can help to shift, mitigate, or question some of the systems and power hierarchies prevalent in global health research.

Mutual capacity building is, in many ways, a way of working and attitude toward learning and partnership that is challenging to capture in a set of steps. However, having a specific set of steps can make this concept more actionable, provide guidance for groups that want to work together or funders that aim to support bidirectional projects. While this work draws on our experience in mutual capacity building, the process has not been tested in its entirety and will require additional testing and refinement. Our goal in proposing MCB-MA was to contribute to an ongoing conversation, rather than provide the final word.

MCB-MA should be further evaluated in future work and compared with less structured processes for collaborative intervention adaptation. There has been little prior empirical or theoretical work on how to evaluate or define success in mutual capacity building. Most distally, mutual capacity building could improve clinical outcomes by facilitating shared knowledge and iterative improvement of the intervention and its implementation. These clinical outcome changes, however, may be difficult to evaluate, as they may be small effects and result over a longer time. Mutual capacity building is primarily a process change that aims to increase equity and inclusion in the way that research is conducted, which may be best evaluated by examining team outcomes (e.g., longevity of collaboration, parity in funding or promotions, or perception of psychological safety within the team). Finally, the implementation of the MCB-MA model itself could be evaluated by assessing the feasibility of each step, relative cost of this more involved process, whether team members like the process (acceptability) or feel it meets their needs (appropriateness) [52], and the reach of MCB-MA (for instance, has use extended beyond a small number of well-resourced academic centers?) [53]. Selecting outcomes to evaluate, however, is complicated by the multiple, potentially competing, goals of multi-site partnership. Each group of collaborators who undertake this process will come from a different socioeconomic and cultural context and face a different set of health system needs and resources. Groups may weigh differently how much they prioritize, for instance, equity within the team, receiving ongoing funding to facilitate partnership longevity, or public health impact from the intervention on which the team works. While these priorities could be mutually reinforcing, they are often in tension.

There are numerous barriers that prevent bidirectional, mutually beneficial research partnerships from forming and being sustained. These limit the feasibility of implementing MCB-MA but also underscore the importance of having a model such as this. First, often unintentional, yet nevertheless supremacist attitudes of white, wealthy, HIC-based leaders have long discounted LMIC knowledge, practices, and ways of approaching problems [54]. These attitudes may limit the openness of HIC practitioners and researchers to learn from collaborators in other settings [11, 54]. Biases, however, can work bidirectionally, with LMIC-based leaders perceiving HIC collaborators as condescending, overly removed, or only a source of funding. Second, many funding structures are set up to support either projects based in HICs, often in the country where the funder is based (“community health”) or international projects in LMICs (“global health”) [14], not projects that cut across settings. Funders may also not be willing to invest in the process of building a partnership, without promise of tangible deliverables. Lack of funding for robust partnership building processes may make the activities in Phase 1 of MCB-MA very challenging. However, having a model like MCB-MA may help structure and provide a theoretical basis for how teams approach seeking such funding. Third, building deep and respectful partnerships is challenging and a time-intensive investment, particularly across a vast distance, numerous time zones, and different cultures. Partners are often balancing time-constrained schedules, competing priorities, and lack funding to support initial conversations [11]. MCB-MA cannot address all of these challenges, but it does provide researchers and implementers with some pragmatic guidance that can structure their initial efforts to overcome these barriers.

MCB-MA is a model for centering equity and collaboration within research processes—a scope that has inherent limitations. It challenges ways of working within a single project but does not directly challenge the broader hierarchies and inequities in research or development fields or broader social and political systems in which we work. In his seminal writings, Brazilian scholar and activist Paolo Freire (Supplementary Table 1) places his deconstruction of the power relations between student and teacher necessarily within a broader vision of social change. Breaking down the micro-hierarchies within existing systems is not meaningful without concurrently working to deconstruct the broader structural violence that led to these systems and hierarchies [55, 56]. This procedure does not necessitate advocacy or other work that challenges structural inequities between countries, regions, or cities or the inequities created by existing research funding structures and academic hierarchies. It also does not directly address social forces including sexism, racism, or ableism. This is both a limitation of this procedure and an opportunity to embed it within existing efforts for structural activism and social change.

Our approach has several limitations. First, we drew from several different cases of mutual capacity building to illustrate the steps of MCB-MA. None of these cases, however, used the full MCB-MA model. Rather, our experiences in mutual capacity building allowed us to test components of this process and identify activities that facilitated shared learning, which we incorporated into the full model. Evaluation of implementation outcomes, particularly the feasibility and acceptability of the MCB-MA model within diverse research and practitioner teams, is essential. Second, we drew primarily on experiences working between HICs and LMICs and from experience working within behavioral health to inform the development of MCB-MA. This procedure, however, could also be used between two settings within an HIC or LMIC or between an upper-middle income country and a low-income country. It could also be used in fields outside of behavioral health [57]. Third, MCB-MA is potentially more resource-intensive than approaches to intervention adaptation that have less intentional focus on partnership building and mutual feedback. There may be a trade-off between putting resources toward this foundational partnership building and toward scaling an intervention. This trade-off is important to acknowledge and may make all or some parts of MCB-MA less useful in some situations, including humanitarian contexts [58], where rapid adaptation may be imperative. However, even in these cases, we hope the values shared in MCB-MA can provide a useful framework for approaching mutual capacity building.

## Conclusions

MCB-MA is among the first models to define a process for mutual capacity building. The steps outlined contribute to the ongoing discourse around how to engage in shared learning across populations and sites. This procedure requires further implementation, evaluation, and iterative improvement based on lessons learned from its use in different sites. Use of this procedure should go together with greater efforts to develop cultural humility or critical consciousness training, particularly for HIC collaborators. Changing the ways in which we work is an essential part to making the field of global health more equitable. Facilitating opportunities for more equitable collaboration and LMIC leadership is a change that could have far reaching clinical and policy impacts.

### Supplementary Information

Supplementary Material 1.

## Data Availability

Not applicable.

## Abbreviations

ADAPT-ITT: Assessment, Decision, Adaptation, Production, Topical experts, Integration, Training, and Testing
HICs: High-income countries
LMICs: Low- and middle-income countries
MCB-MA: Mutual Capacity Building Model for Adaptation
mhCACI: Mental health Cultural Adaptation and Contextualization for Implementation
