# Supplementary material for: Mutual capacity building model for adaptation (MCB-MA): a seven-step procedure for bidirectional learning and support during intervention adaptation
Source: Glob Health Res Policy. 2024 Jul 2;9:25. doi: 10.1186/s41256-024-00369-8 (PMC11218126; doi:10.1186/s41256-024-00369-8)
Supplement: Supplementary file 1 — Supplementary Material 1. [file 41256_2024_369_MOESM1_ESM.docx]

**Supplemental Table 1. Theories and models informing MCB-MA**

| **Theory** | **Description** | **Contribution to mutual capacity building** |
| --- | --- | --- |
| **Community-based participatory research (CBPR)** | CBPR prioritizes involving people from the communities where research will take place as key decisionmakers throughout the research process (1). Often in CBPR, researchers provide training for community members on the research process, while researchers learn about the values and needs of the community from community members (2). | CBPR emphasizes iterative, long-term partnership building; sharing of findings between partners; and mutual learning (1).  One CBPR model includes a “Partnering processes” domain, which made up of three mutually reinforcing sub-domains: individual characteristics of those involved, relationships between those involved, and partnership structures that act as parameters on the partnerships. This domain centers on the importance of relationship building with an emphasis on negotiating power relationships and having people from the community where the research will take place be included in all decisions. It also underscores the importance of developing formal shared agreements to govern the partnership (3).  While CBPR certainly involves bidirectional learning, the focus remains on a single setting (often the lower resource setting) rather than on clear bidirectional benefit and implementation across settings. Additionally, CBPR typically focuses on partnerships between researchers and service users, rather than two groups of researchers (4), which creates different types of power dynamics (5). |
| **Theory of change** | Using the Theory of Change model, key stakeholders meet to determine the desired outcome of an intervention or evaluation then work backwards to come up with the process and mechanisms through which that outcome can be achieved (6). This is a common approach for the development and evaluation of public health interventions (7), and it can be applied to nearly any type of project. | Theory of Change does not have specific mechanisms for bidirectional projects. Similarly, Theory of Change does not specify who is involved in the iterative meetings and can vary widely in how inclusive versus elite driven it is (8). |
| **Freirean participatory dialogue** | Outlined in his seminal work *The Pedagogy of the Oppressed*, Brazilian activist and scholar Paolo Freire outlines an approach to social power dynamics that has been used widely in education and informed the development of CBPR (9). Most concretely, Freire argues the student-teacher hierarchy must be broken down and shift from a teacher imparting knowledge to a student to a dialogue between teacher and student, a “knowledge democracy” (10). | While this philosophy has been used extensively in both education and public health (11, 12), its use in isolation fails to acknowledge a key tenant of Freire: education takes place within a broader social and political system, and Freire advocates for radical deconstruction of the power relations and systems of violence that lead to systemic oppression. He places his philosophy of education within a much broader vision of social transformation (10, 12). Scholars of Freire have critiqued those who use Freire’s teaching techniques without also incorporating his challenging, revolutionary politics (12).  Mutual capacity building could be placed in the same light: engaging in bidirectional learning between a high-resource and low-resource setting without questioning and working to dismantle the broader power relations and systems of violence that created resource differences is missing a fundamental aspect of this work. |
| **Implementation frameworks – Adaptome and “Public health program capacity for sustainability framework”** | Adaptation of interventions for new settings is a core tool of implementation science to facilitate feasibility, acceptability, and appropriateness in the new setting (13, 14). While many implementation theories and frameworks have been developed around adaptation, we highlight two particularly relevant frameworks.  **Adaptome:** Implementation science emphasizes the rapid dissemination of evidence-based interventions and implementation strategies, which can include facilitating sharing of ideas amongst researchers and implementers. The Adaptome, for instance, is proposed public data repository in which both researchers and real-world implementers could iteratively share experiences and data on the adaptation and implementation of behavioral interventions to speed and improve adaptation processes (15).  **Public health program capacity for sustainability framework:** Sustainability of interventions is a key outcome in implementation science (14). While there are myriad sustainability models within that field, one predominant one is the “Public health program capacity for sustainability framework.” This framework’s creators combined literature on sustainability with a structured survey of public health experts to develop the nine domains that they posit are important for the sustainability of interventions. They divide domains into those that are controlled by the team running the project (organizational capacity, program adaptation, program evaluation, communications) and those that are not controlled by the project team (funding stability, political support, partnerships, public health impacts) (16). | Broadly, the learning from mutual capacity building may, particularly for the originating group, focus on implementation.  **Adaptome:** Transparency about processes is a core value of both the Adaptome and mutual capacity building. The Adaptome is proposed as a third-party repository and, so learning could involve many more stakeholders than just two partners. However, it does not focus on relationship building or partnership formation, only on exchange of information.    **Public health program capacity for sustainability framework:** Sustainability is one of the most challenging aspects of implementation and relatively infrequently examined in the field of implementation science (17, 18). Even if the originating group has successfully implemented the intervention at scale, this model emphasizes that sustainability is a process, not a concrete outcome. Sustainability is an aspect of implementation where engaging in mutual learning with the adapting site may allow the originating site to innovate, improve, and recommit to the intervention’s sustainability (16). |
| **Projeto Interdisciplinar do Sexualidade, Saúde Mental e Aids (PRISSMA)** | PRISSMA modeled the adaptation of an HIV prevention intervention that was developed in the US for use in people with mental health conditions in Brazil. The team’s approach to adaptation was to embed within the new culture, taking an ethnographic approach. To create a shared understanding across the team, they first had a training in English and Portuguese for team members from both settings on the local cultural and context in Brazil and core research methods. Their adaptation process also involved developing a community advisory board to inform the adaptation process; working to build team unity across the diverse research team through both informal social events and more structured exercises; completing one year of ethnographic work in the new setting including observation, interviews, and focus groups; and engaging local mental health care providers in testing and iteratively adapting the intervention through a series of workshops with the research team (19). | In the PRISSMA model, investigators prioritized relationships within the team doing the adaptation and deep understanding of the culture in the new setting. PRISSMA provides an example of concrete steps for building unity within a cross-cultural research team and emphasizes the importance of this team unity for efficient and effective adaptation, informing a step in in MCB-MA. PRISSMA also focuses on building understanding of the local context and culture to inform the intervention’s adaptation and engaging a wide range of local stakeholders directly in adaptation. This inclusive, culturally centered approach centers equity, a value that should inform any approach to mutual capacity building. PRISSMA, however, focuses primarily on adaptation in one LMIC setting. While the US team almost certainly took back lessons to inform their implementation of the intervention in the US, these were not made explicit nor was there a specific process for developing them. |

**References**

1. Israel BA, Schulz AJ, Parker EA, Becker AB. Review of community-based research: assessing partnership approaches to improve public health. Annu Rev Public Health. 1998;19:173-202.

2. Harris DA, Pensa MA, Redlich CA, Pisani MA, Rosenthal MS. Community-based Participatory Research Is Needed to Address Pulmonary Health Disparities. Ann Am Thorac Soc. 2016;13(8):1231-8.

3. Oetzel JG, Boursaw B, Magarati M, Dickson E, Sanchez-Youngman S, Morales L, et al. Exploring theoretical mechanisms of community-engaged research: a multilevel cross-sectional national study of structural and relational practices in community-academic partnerships. Int J Equity Health. 2022;21(1):59.

4. Julian McFarlane S, Occa A, Peng W, Awonuga O, Morgan SE. Community-Based Participatory Research (CBPR) to Enhance Participation of Racial/Ethnic Minorities in Clinical Trials: A 10-Year Systematic Review. Health Commun. 2022;37(9):1075-92.

5. Damon W, Callon C, Wiebe L, Small W, Kerr T, McNeil R. Community-based participatory research in a heavily researched inner city neighbourhood: Perspectives of people who use drugs on their experiences as peer researchers. Social science & medicine (1982). 2017;176:85-92.

6. Weiss CH. Nothing as practical as good theory: Exploring theory-based evaluation for comprehensive community initiatives for children and families. New approaches to evaluating community initiatives: Concepts, methods, and contexts. 1995;1:65-92.

7. Breuer E, Lee L, De Silva M, Lund C. Using theory of change to design and evaluate public health interventions: a systematic review. Implement Sci. 2016;11:63.

8. Sullivan H, Stewart M. Who owns the theory of change? Evaluation. 2006;12(2):179-99.

9. Freire P. Pedagogy of the oppressed: Bloomsbury publishing USA; 2018.

10. Wallerstein N, Giatti LL, Bógus CM, Akerman M, Jacobi PR, De Toledo RF, et al. Shared participatory research principles and methodologies: perspectives from the USA and Brazil—45 years after Paulo Freire’s “pedagogy of the oppressed”. Societies. 2017;7(2):6.

11. Brouse CH, Basch CE, Wolf RL. Using concepts from Freire’s pedagogy of the oppressed to promote colorectal cancer screening in an urban minority population. Journal of Health Disparities Research and Practice. 2010;4(1):7.

12. McKenna B. Paulo Freire’s blunt challenge to anthropology: Create a pedagogy of the oppressed for your times. Critique of Anthropology. 2013;33(4):447-75.

13. Cabassa LJ, Baumann AA. A two-way street: bridging implementation science and cultural adaptations of mental health treatments. Implement Sci. 2013;8:90.

14. Proctor E, Silmere H, Raghavan R, Hovmand P, Aarons G, Bunger A, et al. Outcomes for implementation research: conceptual distinctions, measurement challenges, and research agenda. Adm Policy Ment Health. 2011;38(2):65-76.

15. Chambers DA, Norton WE. The Adaptome: Advancing the Science of Intervention Adaptation. American journal of preventive medicine. 2016;51(4 Suppl 2):S124-31.

16. Schell SF, Luke DA, Schooley MW, Elliott MB, Herbers SH, Mueller NB, et al. Public health program capacity for sustainability: a new framework. Implement Sci. 2013;8:15.

17. Rose AL, Jack HE, Wan C, Toloza E, Bhattiprolu K, Ragunathan M, et al. Implementing Task-Shared Child and Adolescent Psychological Interventions in Low- and Middle-Income Countries: A Scoping Review. J Clin Child Adolesc Psychol. 2022:1-16.

18. Kemp CG, Weiner BJ, Sherr KH, Kupfer LE, Cherutich PK, Wilson D, et al. Implementation science for integration of HIV and non-communicable disease services in sub-Saharan Africa: a systematic review. AIDS. 2018;32 Suppl 1:S93-S105.

19. Wainberg ML, McKinnon K, Mattos PE, Pinto D, Mann CG, de Oliveira CS, et al. A model for adapting evidence-based behavioral interventions to a new culture: HIV prevention for psychiatric patients in Rio de Janeiro, Brazil. AIDS and behavior. 2007;11(6):872-83.
